# Peptide Terminated Polymer Brush Nanomaterials for Label‐Free Separation of Cancer Cells Based on Relative Adhesion

**DOI:** 10.1002/smll.75311

**Published:** 2026-08-19

**Authors:** Stephen Horan, Anita Wdowicz, Nathan Feely, Peng Li, Oya Tagit, Wenxin Wang, Gil U Lee

**Affiliations:** ^1^ School of Chemistry and Conway Institute for Biomolecular and Biomedical Research University College Dublin Dublin Republic of Ireland; ^2^ Magnostics Ltd, Co. Dublin Republic of Ireland; ^3^ Charles Institute University College Dublin Dublin Republic of Ireland

**Keywords:** atom transfer radical polymerization, cell separations, integrins, microfluidics, polymer brushes, RGD

## Abstract

Separation of cell types from complex tissue‐derived populations is a priority goal in biomedical research with direct implications for medical diagnostics and bioprocessing. Dense poly(ethylene glycol) methyl ether methacrylate (PEGMEMA) brush films synthesized by surface‐initiated atom transfer radical polymerization yield a linear growth rate of 0.83 nm min^−^^1^ and thicknesses up to 120 nm. Quartz crystal microbalance with dissipation monitoring confirms suppression of non‐specific protein adsorption from fetal bovine serum. Terminal azide functionalization followed by strain‐promoted copper‐free click chemistry enables immobilization of linear Arg‐Gly‐Asp (linRGD) and cyclic (cRGD) peptides at independently tunable densities, a design space of brush thickness, peptide density, and RGD conformation not previously achieved in microfluidics. Integration into polydimethylsiloxane microfluidic channels with a progressive force ramp (0–25 dynes cm^−^^2^) achieves selective displacement of A375 melanoma from A2780 ovarian cancer cells with 88 ± 15% recovery and >93% viability. Differential focal adhesion maturation underlies separation: A375 cells express 29.7% lower α5β1 integrin density and form nascent adhesions retracting at 120 min (actin falls 37%), whereas A2780 cells develop mature focal adhesions with 92% and 125% increases in talin and actin. This platform transduces integrin profiles and cytoskeletal competence into measurable adhesion phenotypes, enabling precise, gentle label‐free cell separation.

## Graphical abstract

Horan et al. demonstrate that RGD peptide‐functionalized poly(ethylene glycol) brush films synthesized in polydimethylsiloxane microfluidic channels by surface‐initiated atom transfer radical polymerization enable label‐free separation of melanoma and ovarian cancer cells based on differential integrin expression. Confocal microscopy reveals that distinct focal adhesion maturation under progressive hydrodynamic shear governs selective displacement, achieving 88% recovery with greater than 93% viability.

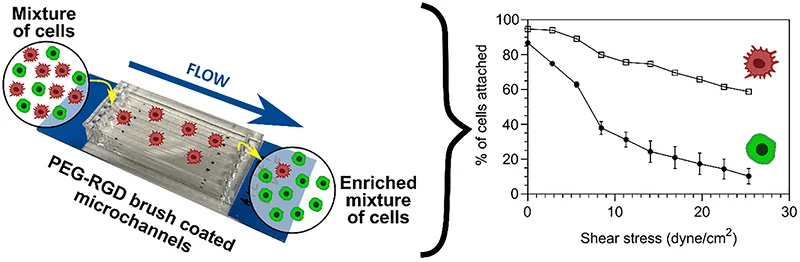

## 1 Introduction

The separation of specific cell types from samples containing heterogeneous cell populations is of critical importance for several fields of biomedical research including medical diagnostics, cancer research and bioprocessing [1]. Fluorescence‐activated cell sorting (FACS) and magnetic‐activated cell sorting devices are recognized as the standard for efficient isolation of desired cell types from heterogeneous mixtures. Both techniques depend on cell‐specific antibody labeling; however, introducing cost, contamination risk, and pre‐treatment steps that can alter cell biology, particularly for cancer cells that have undergone epithelial‐to‐mesenchymal transition (EMT) and downregulate surface antigens such as the epithelial cell adhesion molecule (EpCAM) [2, 3, 4]. Microfluidic (MF) platforms offer label‐free alternatives that exploit intrinsic biophysical differences, i.e., cell size, density, deformability, and polarizability [5], using micro‐filters [6, 7], micro‐post arrays [8, 9, 10], branched channels [11], acoustophoresis [12, 13, 14], and dielectrophoretic fields [5, 15]. While powerful, the complex geometries required impose fabrication demands that constrain cost and wider clinical adoption [3, 16].

Affinity‐based cell separations in MF channels functionalized with antibodies or key cell‐binding proteins such as fibronectin (FN) can offer potential solutions to the issues of cost and scalability in cell separation [17, 18, 19, 20]. FN is an extracellular matrix (ECM) glycoprotein that plays a major role in cell adhesion and mobility through interactions between membrane adhesion proteins called integrins and arg‐gly‐asp (RGD) motifs present in FN [21, 22]. While the preparation of FN‐functionalized surfaces is relatively simple, the density and orientation of RGD cannot be defined well for the FN‐functionalized surfaces, which presents a challenge for controlling integrin‐mediated specific cell binding [23].

Polymer brushes, dense layers of surface‐tethered chains synthesized from surface‐bound initiators, resolve these limitations by providing a non‐fouling background on which bioactive ligands can be presented at precisely defined surface densities [24, 25, 26, 27]. Poly(ethylene glycol) (PEG) brushes prepared by surface‐initiated atom transfer radical polymerization (SI‐ATRP) suppress non‐specific protein adsorption through steric repulsion and strong surface hydration, establishing a well‐defined baseline for specific integrin–ligand interactions [27]. End‐group functionalization with RGD peptides via click chemistry enables integrin‐mediated cell adhesion at densities not achievable on adsorbed protein coatings [28, 29]. The binding activity and integrin subtype selectivity of RGD are critically dependent on peptide conformation: cRGD, conformationally constrained by head‐to‐tail cyclization, exhibits significantly higher affinity for αv‐class integrins than linRGD, which preferentially engages α5β1 [30, 31]. Despite this knowledge, the integration of SI‐ATRP‐derived non‐fouling PEG brush films bearing conformation‐specific RGD end‐groups within a MF cell separation platform has not previously been reported [32, 33, 34, 35, 36].

Here, dense PEGMEMA brush films are synthesized on silica surfaces by activator regenerated by electron transfer SI‐ATRP (SI‐ARGET‐ATRP) and end‐functionalized with linRGD and cRGD via strain‐promoted copper‐free click chemistry, providing independent control over brush thickness, peptide surface density, and RGD conformation within a PDMS MF channel. Application of a progressive hydrodynamic force ramp (0–25 dynes cm^−^^2^) achieves label‐free separation of A375 melanoma from A2780 ovarian cancer cells with 88±15% recovery and >93% viability. Confocal laser scanning microscopy (CLSM) of talin and actin and flow cytometric integrin profiling reveal that differential focal adhesion maturation, governed by the significantly lower α5β1 integrin expression of A375 cells, provides the biomechanical basis for this antibody‐independent separation [36, 37]. The novelty of the present work lies in integrating RGD‐functionalized brushes of tunable ligand density into a microfluidic device that actively separates and recovers viable cell subpopulations on the basis of differential integrin‐mediated adhesion. To our knowledge, this is the first non‐fouling polymer‐brush interface configured for preparative, label‐ and antibody‐free cell separation under flow, here resolving A375 melanoma from A2780 ovarian carcinoma cells while preserving high viability and recovery.

## 2 Results and Discussion

### 2.1 Synthesis and Characterization of Poly(PEGMEMA) Polymer Brush Films

The polymer brush‐functionalized glass coverslips were prepared via SI‐ATRP utilizing 2‐bromo‐2‐methyl‐(N‐3‐(triethoxysilyl)propyl) propanamide (BiB‐APTES) as a silane‐graftable initiator. The synthesis of BiB‐APTES was performed as described by Panzarasa et al., yielding a product with ^1^H NMR and FTIR spectra consistent with previous reports (Experimental, Synthesis of ATRP macroinitiator) [32]. The deposition of BiB‐APTES on clean borosilicate glass coverslips proceeded by a hydrolysis‐condensation reaction in THF with a catalytic amount of acetic acid (Figure 1A). Immobilization of the BiB‐APTES initiator was confirmed by ellipsometric detection of a new layer with a thickness of 0.78 ± 0.10 nm, accompanied by an increase in the static water contact angle from <5° to 52.3° ± 0.81°. These observations are consistent with previously reported BiB‐APTES‐functionalized silica substrates and confirm successful initiator immobilization [33].

**FIGURE 1:**
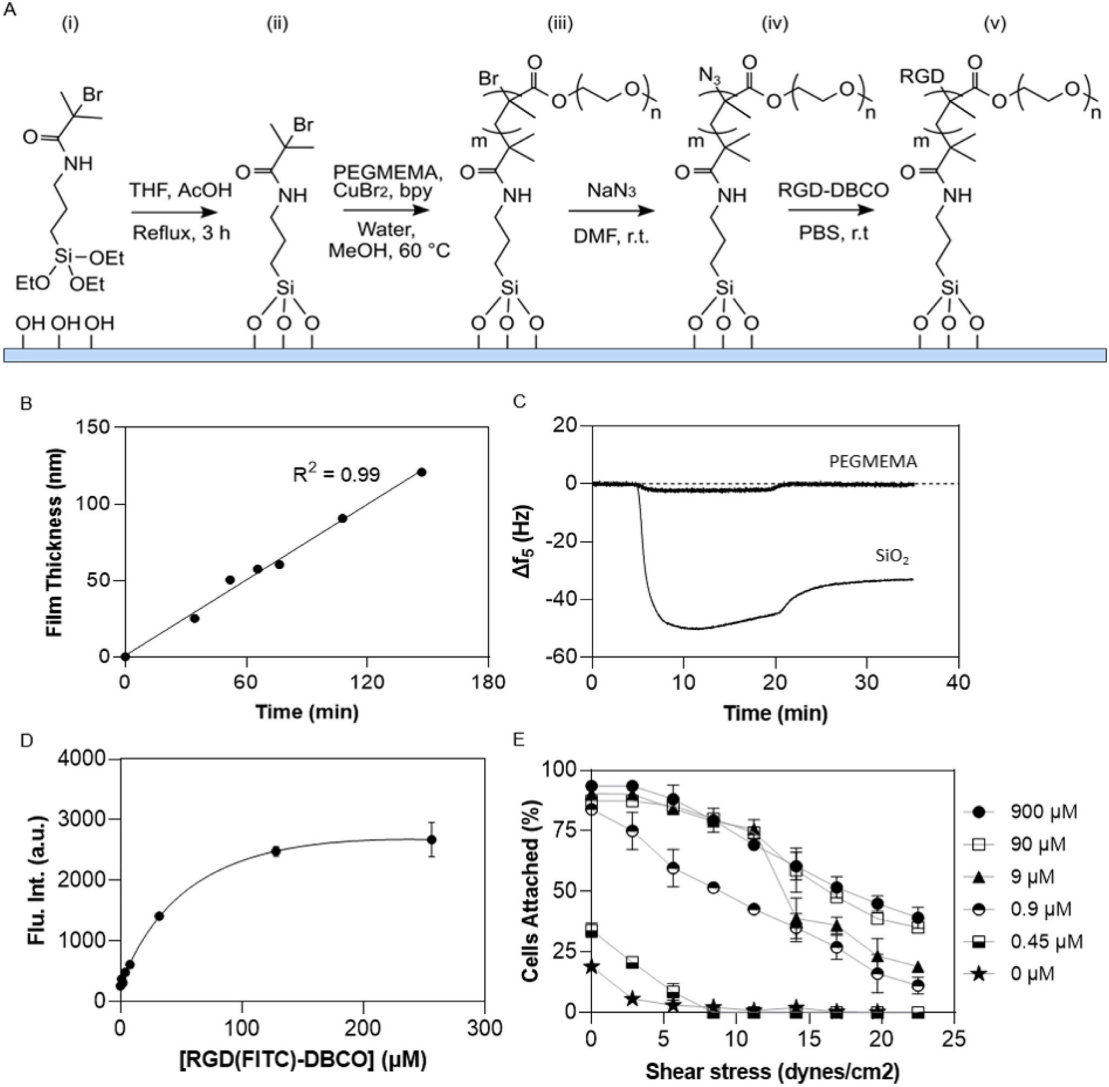
Synthesis and characterization of the nanoscale polymer brush films. (A) Reaction scheme illustrating the synthetic pathway to RGD‐functionalized polymer brushes. (i) Immobilization of BiB‐APTES initiator through condensation reaction onto silica substrates. (ii) Formation of polymer brush films by ATRP of PEGMEMA from BiB‐APTES functionalized substrates. (iii) Conversion of the terminal bromide group to azide by nucleophilic substitution with sodium azide. (iv) Conjugation of DBCO‐functionalized peptide to polymer brush via 1,3‐dipolar cycloaddition to yield (v) RGD‐functionalized polymer brush films. (B) The dry thickness of poly(PEGMEMA) brush films following different polymerization times measured using spectroscopic ellipsometry. Linear regression analysis is presented with R^2^ = 0.99. (C)  Quartz crystal microbalance with dissipation monitoring analysis of adsorption of 1% FBS solution to sensors which were unmodified (SiO_2_) and functionalized with PEGMEMA polymer brush films. Changes in the 5th harmonic resonance frequency are presented. (D) Fluorescence intensity measured using confocal microscopy of PEGMEMA‐N_3_ polymer brushes following incubation with increasing concentrations of RGDK(FITC)‐PEG_4_‐DBCO. Mean fluorescence intensity (n = 3) is presented with SD. (E) Analysis of A375 cell binding and displacement from 70 nm linRGD‐PEGMEMA polymer brush films prepared using increasing concentrations of linRGD‐PEG_4_‐DBCO from 0 µM to 900 µM.

Polymer brush films were synthesized using ARGET‐ATRP to achieve controlled polymerization of PEGMEMA in an aqueous solution with copper(II) bromide and 2,2′‐bipyridine as a catalyst (Figure 1A). Living polymerization characteristics were ensured by adding 0.5 molar equivalents of sodium ascorbate (reducing agent) relative to the catalyst, yielding a linear brush growth rate at a suitable reaction rate. Figure 1B presents ellipsometry measurements of dry polymer brush films grown under these conditions. A linear growth rate of 0.83 nm min^−1^ was observed for films with dry thicknesses up to 120 nm, accompanied by a decrease in water contact angle to 41.3° ± 0.53°, consistent with the hydrophilic character of PEG brush films [34]. Linear regression of the kinetic plot yielded R^2^ = 0.99, confirming high reproducibility of film thickness control.

The non‐specific binding of proteins to poly(PEGMEMA) brushes was analyzed by quartz crystal microbalance with dissipation (QCM‐D) monitoring experiments. Quartz QCM‐D sensors were functionalized with a 60–80 nm poly(PEGMEMA) brush film as described above. After stabilizing the sensors in PBS, they were exposed to a 1% fetal bovine serum (FBS) solution for 15 min before re‐stabilizing the sensors in PBS. Figure 1C presents the change in the fifth harmonic frequency (Δf_5_) of bare and polymer brush‐modified sensors following exposure to the FBS solution. Bare SiO_2_ sensors exhibited a frequency drop of 33.0 Hz, indicating protein adsorption.

The adsorbed protein mass density (*Δm*) has been reported using the Sauerbrey equation, where *Δm* is dependent on the mass sensitivity constant (C = 17.7 ng cm^−2^ Hz^−1^), the frequency shift (*Δf*), and the harmonic overtone (*n*):

$$\Delta m=-C\frac{\Delta f}{n}$$

The density of protein adsorbed on the bare sensor was thereby calculated to be 117 ng cm^−2^. In contrast, no detectable frequency shift was observed for poly (PEGMEMA)‐functionalized sensors following FBS exposure, indicating no detectable non‐specific protein adsorption (within the sensitivity of the QCM‐D measurement).

### 2.2 Facilitating Cellular Adhesion and Displacement from Polymer Brush Films with RGD

The immobilization of the cell‐binding motif, RGD, on non‐fouling polymer brush films is critically important to facilitate cellular adhesion to the polymer brushes. The poly(PEGMEMA) brushes were first modified by replacing the terminal bromide group with an azide group in an S_N_2 substitution reaction with sodium azide (Figure 1A) [38, 39, 40]. RGD was then immobilized by reacting the azide‐functionalized brushes (dry thickness 60–80 nm) with serial dilutions of a fluorescently labelled RGD peptide coupled to DBCO through a flexible four‐unit PEG spacer (RGD(FITC)‐PEG_4_‐DBCO) via the strain‐promoted, copper‐free azide–alkyne cycloaddition. The conjugation was carried out under continuous flow. The reaction is limited by the interfacial reaction rather than by mass transport of the DBCO conjugate to the surface because the strain‐promoted cycloaddition is intrinsically slow, with a second‐order rate constant of ∼1 M^−^^1^ s^−^^1^ in aqueous buffer that is largely insensitive to buffer identity and ionic strength [41, 42]. The continuous flow maintains an effectively constant, uniform reagent concentration along the channel (pseudo‐zero‐order in DBCO), so that coverage is set reproducibly by the bulk concentration and reaction time and is uniform across the film. This flow‐based protocol gave highly reproducible RGD coverage from film to film.

Fluorescence intensity of the polymer brush film interface was then quantified using CLSM and image analysis in ImageJ to extract mean fluorescence intensity per unit area. Figure 1D shows fluorescence intensity as a function of peptide conjugate concentration. Intensity increased before reaching a plateau at 128 µM RGDK(FITC)‐PEG_4_‐DBCO. We interpret the fluorescence signal as a *qualitative* measure of RGD coverage rather than an absolute density, because at the high local fluorophore densities approached here, homo‐FRET self‐quenching between neighboring FITC labels (Förster radius R_0_ ≈ 4.5 nm) is expected to attenuate the emitted signal well before true steric saturation, which on steric grounds corresponds to a mean inter‐ligand spacing of order 1–2 nm. The plateau therefore does not by itself establish a close‐packed monolayer, and we do not derive an absolute areal density from it; it does, however, demonstrate reproducibly that RGD coverage rises monotonically with, and can be controlled by, the conjugation‐solution concentration near 128 µM.

The effect of RGD density on cell adhesion was examined by incubating A375 cells on brush films (60–80 nm dry thickness) in microfluidic channels functionalized with linRGD‐DBCO conjugate solutions ranging from 0 to 900 µM. Adhesion strength was assessed by cell displacement following a 90 min incubation and exposure to shear flow. Figure 1E shows cell displacement characteristics as a function of linRGD concentration. Adhesion of A375 melanoma cells was strongly dependent on linRGD density, proportional to the linRGD‐DBCO conjugate concentration used. Brushes functionalized with 900 and 90 µM linRGD‐DBCO yielded near‐saturated surfaces with similar displacement profiles; 39% ± 4.3% and 35% ± 1.7% of cells remained attached, respectively, at the end of the force ramp protocol. Brushes functionalized with 9, 0.9, and 0.45 µM linRGD‐DBCO showed progressively greater detachment, with 19% ± 0.45%, 11% ± 3.4%, and 0% ± 3.2% of cells remaining attached, respectively. Unmodified brush films yielded near‐complete cell detachment at the lowest applied shear stress of 2.8 dynes cm^−2^, confirming the non‐fouling character of the PEGMEMA film. These results indicate that specific RGD–integrin interactions mediate adhesion to linRGD‐functionalized brush films, with adhesion strength tunable through surface RGD density.

### 2.3 Tuning Cellular Adhesion to RGD‐Functionalized Polymer Brush Films

Integrins display a wide range of binding affinities for different RGD conformations, with cyclic RGD (cRGD) emerging as a more potent integrin‐binding ligand than its linear counterpart [30, 31]. Figure 2A presents displacement characteristics of A375 and A2780 cells from brush films functionalized with 0.9 µM linRGD or cRGD following 90 min incubation and a linear shear ramp to 22.5 dynes cm^−2^. High initial adhesion (>84%) was observed for both cell lines on both brush types. In contrast to the substantial displacement observed on linRGD films (>87% of A375 cells displaced), A375 cells strongly resisted displacement from cRGD films (<6.9% detachment). A2780 cells were resistant to displacement on both film types, with 22% and 27% displaced from linRGD and cRGD films, respectively. These results highlight the important role that RGD conformation plays in governing integrin‐mediated adhesion strength.

**FIGURE 2:**
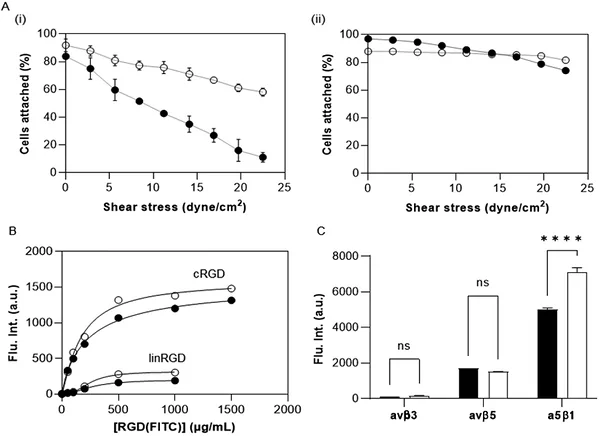
Characterization of cell adhesion and displacement from RGD functionalized polymer brush films. (A) Analysis of the displacement of A375 (●) and A2780 (°) cells from polymer brush film‐modified microfluidic channels functionalized with 0.9 µM (i) linRGD and (ii) cRGD following a 90 min incubation and a force ramp displacement protocol. Mean cell attachment is presented with SD where n>3. (B) Flow cytometry analysis of fluorescently labelled linear and cyclic RGD binding to A375 (●) and A2780 (°) cell lines. The fluorescence intensity per unit area calculated by dividing the median fluorescence intensity by the squared forward scatter intensity is presented. (C) Flow cytometry analysis of integrin expression by A375 (black) and A2780 (white) cells following immunofluorescence staining. The fluorescence intensity per unit area calculated by dividing the median fluorescence intensity by the squared forward scatter intensity is presented. Mean fluorescence intensity is presented with SD where n = 2. Statistical comparisons were performed using a two‐way ANOVA with a Bonferroni post‐hoc test, where ns denotes not significant and ^****^ *p* < 0.0001.

The differential binding affinities of A375 and A2780 cells for linRGD and cRGD were further investigated by flow cytometry. Cells were incubated with increasing concentrations of fluorescently labeled linRGD and cRGD, and fluorescence intensity per unit area was quantified by normalizing the median fluorescence intensity (MFI) by the square of forward scatter intensity (proportional to cell radius) to correct for cell size differences. Figure 2B presents RGD binding profiles for both cell lines. Fluorescence intensity increased with peptide concentration, reaching saturation at approximately 500 µg mL^−1^. A375 cells demonstrated 37% and 13% lower saturation MFI for linRGD and cRGD, respectively, compared to A2780 cells. The binding density of cRGD was 6.3‐fold and 4.5‐fold greater than linRGD for A375 and A2780 cells, respectively, consistent with the higher integrin affinity of cRGD [30].

Integrin expression was profiled by immunofluorescence and flow cytometry. Figure 2C presents fluorescence intensity per unit area for αvβ3, αvβ5, and α5β1. No statistically significant difference in αvβ3 or αvβ5 expression was observed between A375 and A2780 cells; however, a statistically significant difference (*p* < 0.0001) in α5β1 expression was found, with A375 cells expressing 29.7% lower α5β1 density than A2780 cells. This difference in α5β1 expression is consistent with the differential adhesion and displacement behavior observed on linRGD‐ and cRGD‐functionalized brush films, given that α5β1 is the principal integrin subtype for linear RGD engagement. Taken together, these results indicate that cellular adhesion strength on PEGMEMA brush films can be tuned through the conformation and surface density of the presented RGD ligand, with linRGD providing selective detachment of low‐α5β1‐expressing cells.

### 2.4 Cellular Mechanical Response to Polymer Brush Films

While integrin expression determines initial cell binding, cellular adhesion is a dynamic biomechanical process driven by the recruitment of cytoplasmic adapter proteins, including talin and actin, which initiate cytoskeletal reorganization and focal adhesion maturation at the integrin–substrate junction. Figure 3 presents CLSM images of fluorescently labeled talin and actin in A375 and A2780 cells following incubation on 0.9 µM linRGD brush films for 60, 90, and 120 min. Both cell lines exhibited clear morphological changes with contrasting responses to the substrate. A375 cells spread progressively up to 90 min, with nascent adhesions (co‐localized talin and actin) visible at the cell leading edge. These results are consistent with the rounded morphology reported for A375 cells on soft PEG hydrogels (elastic modulus 0.6 kPa), where stiffer substrates favor spreading and mature focal adhesion formation [36]. Notably, A375 cells retracted at 120 min, accompanied by a statistically significant 37% decrease in actin expression relative to 60 min (p < 0.05). A2780 cells displayed contrasting behavior, with 92% and 125% increases in talin and actin expression from 60 to 120 min, respectively, and a 76% increase in talin–actin co‐localization intensity. Actin expression in A2780 cells was approximately 2 fold higher than in A375 cells at 90 min, indicating successful cytoskeletal engagement and mature focal adhesion formation.

**FIGURE 3:**
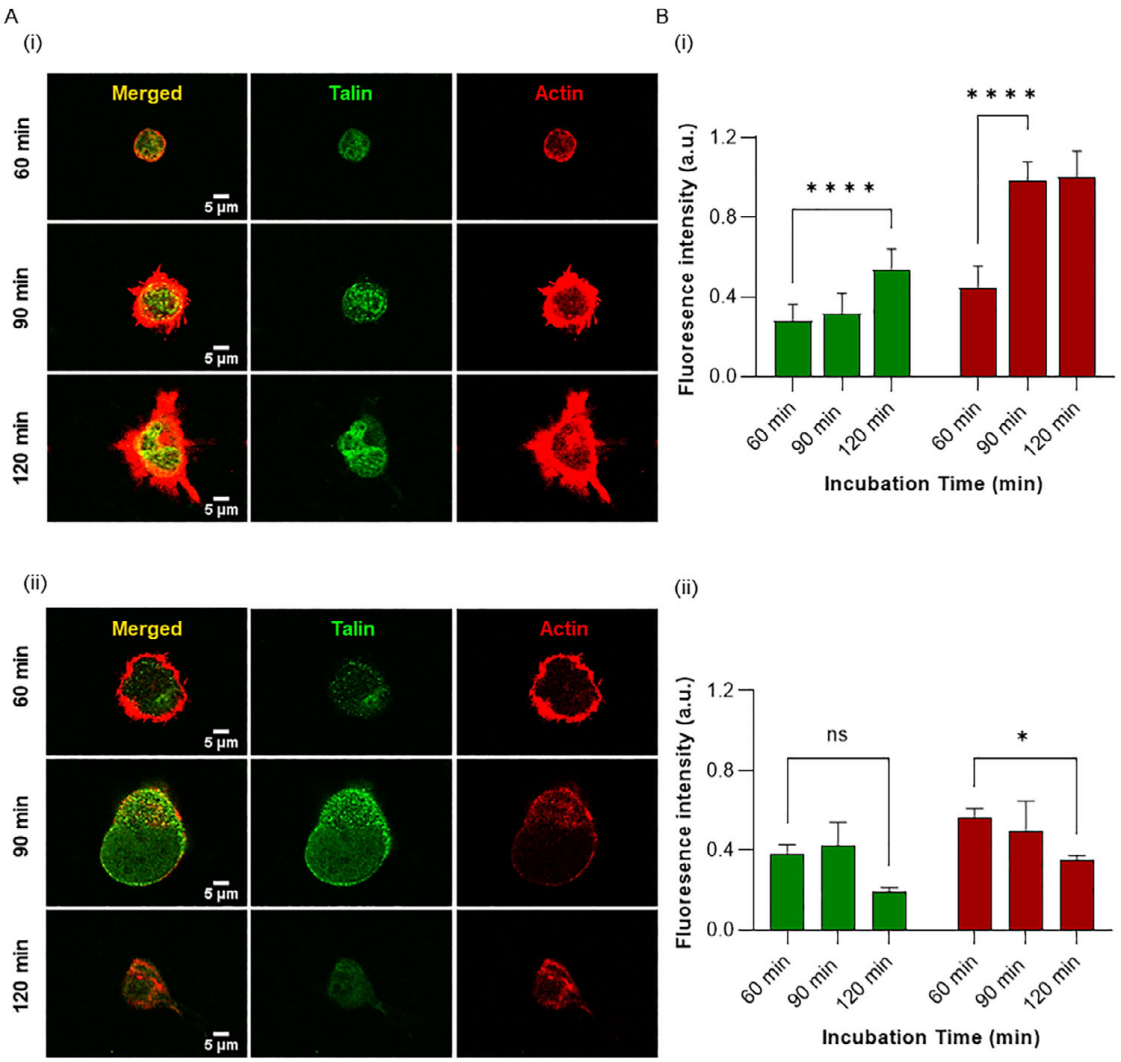
Immunofluorescence staining and confocal microscopy analysis of development of key focal adhesion proteins, talin and actin. (A) CLSM images showing talin and actin channels of (i) A2780 and (ii) A375 cells following incubation on PEG brush films functionalized with 0.9 µM linRGD for different time points. A merged image is presented to highlight co‐localization of focal adhesion proteins appearing in yellow. (B) Fluorescence intensity analysis of talin (green) and actin (red) extracted from CLSM images. The mean fluorescence intensity is presented with SD, n > 5. Statistical comparisons performed by a two‐way ANOVA with Bonferroni post hoc test are presented where ns denotes not significant, ^*^ *p* < 0.05 and ^****^ *p* < 0.0001.

Cell adhesion is initiated by an integrin‐binding event and propagates through mechanosensitive signal transduction involving talin. Upon substrate binding, cellular contractile forces unfold the talin rod domain stochastically, revealing cryptic vinculin‐binding sites and thereby reinforcing the integrin–actin cytoskeleton linkage. A375 cells did not show significant talin or actin recruitment following initial binding to the brush film, resulting in immature focal adhesions and limited adhesion strength. A2780 cells, by contrast, formed mature focal adhesions, consistent with their selective retention on the brush film under the force‐ramp displacement protocol (Figure 2A).

### 2.5 Microfluidic Separation of a Mixed A2780 and A375 Cell Population

Following confirmation of weaker focal adhesions in A375 cells, the selective displacement of A375 cells from A2780 cells in a mixed population was demonstrated using 0.9 µM linRGD brush films (60–80 nm dry thickness). A2780 cells were labeled with Vybrant DiO to enable cell‐line identification during separation. A 1:1 mixture of A375 and labeled A2780 cells (1.5 × 10^6^ mL^−1^) was prepared. Figure 4A presents cell displacement profiles during separation using the force‐ramp protocol. The displacement behavior mirrored single‐cell‐line experiments (Figure 2A). A375 cells were selectively displaced with 88% ± 15% recovery at 25 dynes cm^−2^, while 38% ± 2% of A2780 cells were displaced under the same conditions, yielding an A375 purity of 70% ± 17% in the detached fraction. Cell viability was assessed for single‐population displacements using a trypan blue assay on the channel effluent (Figure 4C). Viability >93% was observed for both cell lines before and after separation, with no statistically significant change, confirming that the applied shear forces were sufficient to enable detachment without inducing cell death.

**FIGURE 4:**
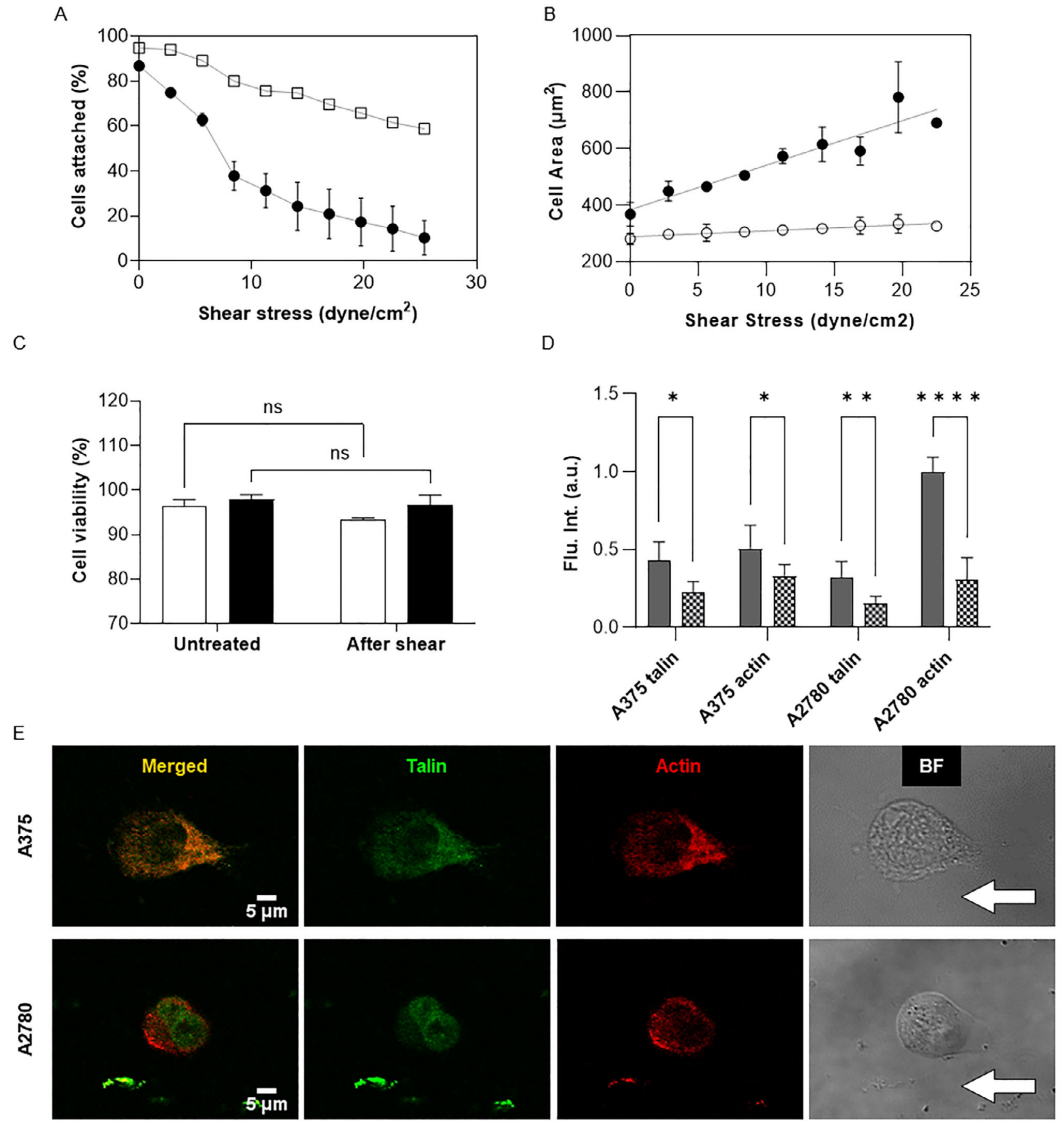
Analysis of mixed cell separation and biomechanical response to applied shear forces. (A) Force‐ramp displacement profiles for a 1:1 A375/A2780 mixed population on 0.9 µM linRGD brush films (mean ± SD, n = 3). (B) Cell area increase of A375 (●) and A2780 (°) cells as a function of applied shear stress (mean ± SD). (C) Trypan blue viability of displaced cells (mean ± SD, n = 3). (D) Mean fluorescence intensity of talin (green) and actin (red) following force‐ramp displacement (± SD, n > 5); ^*^ *p* < 0.05, ^**^ *p* < 0.01, ^****^ *p* < 0.0001, two‐way ANOVA with Bonferroni post‐hoc test. (E) CLSM images (merged, talin, actin, and brightfield) of A375 and A2780 cells following force‐ramp displacement; white arrow indicates the direction of applied shear.

The biomechanical response of cells to applied shear was assessed by measuring cell area changes during the force‐ramp protocol using ImageJ thresholding analysis. Figure 4B shows cell area as a function of shear stress. Cell area increased linearly (R^2^ = 0.99 and 0.96 for A375 and A2780, respectively) with shear up to 22.5 dynes cm^−2^, with A375 cells showing an 87% ± 11% total area increase versus 16% ± 0.5% for A2780 cells. Both cell lines adopted a “teardrop” morphology with elongation parallel to the flow direction [37]. The cytoskeletal basis of these changes was examined by CLSM (Figure 4D,E). Statistically significant reductions in talin and actin expression were observed in both lines: A375 cells showed 46% and 34% reductions in talin and actin, respectively; A2780 cells showed 52% and 70% reductions. The marked reduction in actin expression in A2780 cells correlates with the retraction of filopodia projections observed in Figure 3A, reflecting the dynamic cytoskeletal response to shear. Collectively, the reductions in talin and actin expression across both cell lines indicate that the force‐ramp protocol disrupted surface attachment in both populations, consistent with the observed cell displacement.

## 3 Conclusions

Dense PEGMEMA brush films synthesized by surface‐initiated ARGET‐ATRP and end‐functionalized with linear and cyclic RGD peptides via strain‐promoted copper‐free click chemistry provide a well‐defined, independently tunable platform for integrin‐mediated cancer cell separation in polydimethylsiloxane microfluidic channels. The combination of a non‐fouling brush background, controllable peptide surface density, and programmable RGD conformation constitutes a degree of surface‐design control that we exploit here for label‐ and antibody‐free separation under flow. Application of a progressive hydrodynamic force ramp achieves selective displacement of A375 melanoma cells from A2780 ovarian cancer cells with 88±15% recovery and >93% viability. Confocal laser scanning microscopy of talin and actin, together with flow cytometric integrin profiling, establishes that differential focal adhesion maturation, governed by the 29.7% lower α5β1 integrin density of A375 cells, is the biomechanical basis of separation. Because the mechanism depends on integrin expression and cytoskeletal mechanotransduction rather than on epithelial surface antigens, it is not expected to be affected by the loss of epithelial markers that limits antibody‐based capture—although this expectation remains to be tested directly in a model of epithelial–mesenchymal transition.

We envisage two applications, both of which will require demonstration in real, complex samples before clinical or industrial translation. The first is the enrichment of rare cells from blood, for example candidate circulating tumor cells, that differ from the background cell population in integrin expression and in their response to mechanical stress. Beres et al. classified seven cell types from adhesion‐kinetics data on RGD‐presenting surfaces with accuracies approaching 95%, attributing this discriminating power to the distinct integrin repertoires with which different cell types engage RGD motifs, the same physical basis that underlies the separation reported here [43]. That work is analytical; it assigns a class to each cell in situ but does not recover it. Ours is preparative; it physically isolates and recovers viable populations by differential adhesion but resolves populations rather than classifying individual cells. Because our platform preserves viability, enriched cells could be sequenced and their genetic profile related to the adhesive, integrin‐based phenotype on which they were selected, providing a label‐free route to phenotype‐linked single‐cell genomic analysis. We note that the relevant contaminating population in blood is haematological rather than tissue‐of‐origin epithelium, and that performance against leukocytes and in whole blood remains to be established. The second application is bioprocessing quality control. Most biomanufacturing uses immortalized cell lines, in which genetic and phenotypic drift over successive passages is difficult to monitor; an adhesion‐based method able to detect and remove cells that deviate from the dominant adhesive phenotype could offer a simple means of limiting such drift and maintaining line consistency over time. This technique promises to provide control of cell separation, building on techniques that use polymer brushes [44, 45].

The modular end‐group click chemistry is compatible with any DBCO‐tagged biomolecule, allowing extension to receptor–ligand pairs beyond the integrin–RGD axis, and the planar force‐ramp geometry is compatible with standard photolithography and with channel‐array parallelization to increase throughput without altering the hydrodynamic conditions at the cell surface. Taken together, this work establishes non‐fouling polymer‐brush microfluidics that unite a defined, antifouling surface chemistry with a mechanobiologically grounded separation principle as a foundation for label‐free cell processing and identifies the real‐sample and clinical demonstrations needed to realize it.

## 4 Experimental Section

### 4.1 Materials

All reagents were commercially available and used as received without further purification unless otherwise stated. α‐Bromoisobutyryl bromide, 3‐aminopropyltriethoxysilane, triethylamine, tetrahydrofuran, acetic acid, poly(ethylene glycol) methyl ether methacrylate (M_n_ = 500 g·mol^−1^), copper(II) bromide, 2,2′‐bipyridine, methanol, maleimide‐PEG_4_‐DBCO, dimethyl sulfoxide, sodium azide, accutase, BSA, and goat anti‐talin mAb were purchased from Merck KGaA (Germany). Magnesium sulfate, Triton X‐100, dimethylformamide, and sodium ascorbate were from Acros Organics (USA). RGDC and c(RGDfC) were from Bachem AG (Switzerland); c(RGDK(FITC)C) from Cambridge Peptides Ltd (UK). Sylgard 184 PDMS was from Dowsil (USA). A375 and A2780 cell lines were gifted by Systems Biology Ireland, University College Dublin. RPMI‐1640, FBS, L‐glutamine, penicillin/streptomycin, 16% methanol‐free formaldehyde, Alexa Fluor 568 β‐Phalloidin, and Alexa Fluor 488 anti‐goat and anti‐rabbit mAbs were from ThermoFisher (UK). Rabbit anti‐αvβ3 and anti‐α5β1 mAbs were from Novus Biologicals (UK); rabbit anti‐αvβ5 from Absolute Antibody (UK).

### 4.2 Synthesis of ATRP Macroinitiator (BiB‐APTES)

Triethylamine (10 mL) and 3‐aminopropyltriethoxysilane (14 mL) were dissolved in THF (200 mL), placed on an ice bath, and purged with N_2_ for 5 min. Bromoisobutyryl bromide (9 mL) was added dropwise, yielding a white precipitate. The reaction was warmed to r.t., stirred for 3 h, vacuum‐filtered, the supernatant dried over MgSO_4_, gravity‐filtered, and solvent removed by rotary evaporation to afford BiB‐APTES as an amber oil (21.6 g, 98%). ^1^H NMR (CDCl_3_, 400 MHz) δ 0.64 (m, 2H), 1.22 (t, 8H, J = 6.9 Hz), 1.65 (m, 2H), 1.94 (s, 6H), 3.26 (q, 2H, J = 6.8 Hz), 3.82 (q, 5H, J = 6.9 Hz), 6.85 (br, 1H). FTIR (ATR) ṿmax (cm^−1^): 3350 (NH), 2970–2890 (CH), 1655 (C = O, amide), 1525 (NH), 1445, 1380, 1280, 1165, 1015, 950, 780.

### 4.3 Synthesis of PEGMEMA‐N_3_ Coverslips

Glass coverslips were cleaned by UVO treatment (Jelight 144AX‐220, 20 min) and base piranha (5:1:1 water:ammonia:hydrogen peroxide, 15 min), rinsed with water, and dried with N_2_. Coverslips were refluxed in THF (210 mL) containing AcOH (10 µL) and BiB‐APTES (1 mL) at 78°C for 3 h, cooled to r.t., rinsed with ethanol, dried with N_2_, and placed in a reaction vessel purged with N_2_ (three pump–refill cycles). The ARGET‐ATRP monomer solution was prepared by dissolving PEGMEMA (59.8 g), CuBr_2_ (31.0 mg), and 2,2′‐bipyridine (64.6 mg) in water (107.0 g) and methanol (36.1 g), sparging with N_2_ for 15 min, then adding sodium ascorbate (8.0 mg). The homogeneous red/brown solution was added to the coverslips, and polymerization was conducted at 60°C for the desired time. The reaction was quenched in methanol, and coverslips rinsed with water, then ethanol, and stored at 4°C. Azide end‐groups were introduced by immersion in sodium azide/DMF (4 g L^−1^, 17 h), followed by rinsing with ethanol.

### 4.4 Microfluidic Device Fabrication, Assembly, and RGD Functionalization

Microfluidic channels (6 parallel, 0.2 × 1 × 40 mm) were fabricated by SU‐8 photolithography and cast in PDMS (1:10 catalyst:monomer, 60°C overnight). PEGMEMA‐N_3_ coverslips were masked with PDMS strips, surface‐activated by UVO followed by oxygen plasma (PE‐100, Plasma Etch Inc., USA), and bonded to PDMS channels at r.t. for 30 min. RGDC, c(RGDfC), and c(RGDK(FITC)C) were conjugated to DBCO‐PEG_4_‐maleimide by Michael‐type addition (2:1 RGD:crosslinker, PBS, r.t., 17 h) and stored at 4°C. Channels were rinsed with 0.2 µm filtered PBS, loaded with 300 µL of RGD‐PEG_4_‐DBCO conjugate, recirculated for 17 h in darkness, flushed with PBS, stored under vacuum, and rehydrated before use.

### 4.5 Cell Culture and Labeling

A375 and A2780 cells were cultured in RPMI‐1640 (5% FBS, 1% L‐glutamine, 1% penicillin/streptomycin) at 37°C in 5% CO_2_ and sub‐cultured every 2–3 days. For mixed‐population experiments, A2780 cells were labeled with Vybrant DiO (5 µL per 1 mL of 1 × 10^6^ cells mL^−1^ in serum‐free medium, 37°C, 20 min), recovered by centrifugation, washed twice in serum‐free RPMI, and dye incorporation confirmed by flow cytometry.

### 4.6 Cell Adhesion and Displacement Experiments

Cells were detached with Accutase, resuspended at 1.5 × 10^6^ mL^−1^ in serum‐free RPMI‐1640, and seeded in microfluidic channels on a Zeiss Axio Observer inverted microscope with an environmental chamber at 37°C. Three regions of interest per channel were imaged by time‐lapse microscopy (10 min intervals during adhesion; 10 s during displacement). A syringe pump (KR Analytical, UK) delivered 1× PBS to generate controlled shear stresses, calculated using the planar Poiseuille model $\tau =12(\frac{\mu Q}{wh^{2}})$, where *µ* is the dynamic viscosity, *Q* the flow rate, and *w* and *h* the channel width and height. Cell counts from ImageJ thresholding were used to calculate the percentage of cells adhered after each displacement step relative to the initial seeded count.

### 4.7 Immunofluorescence Staining and Confocal Microscopy

Cells were incubated in RGD‐PEGMEMA channels at 37°C for 60, 90, or 120 min, fixed with 4% paraformaldehyde in PBS (15 min, orbital shaker), and rinsed with PBS. After permeabilization and blocking (0.5% Triton X‐100, PBS/1% BSA), cells were incubated with goat anti‐talin antibody (1:100, 1.5 h, r.t.), then co‐stained with Alexa Fluor 568 β‐Phalloidin (1:200) and Alexa Fluor 488 anti‐goat secondary antibody (1:300) in PBS/1% BSA for 1 h at r.t. Channels were stored at 4°C protected from light. Images were acquired on an Olympus FV1000 confocal microscope (60× oil immersion, fixed acquisition parameters, ≥2 channels per group) and analyzed in ImageJ by threshold masking, particle analysis, and calculation of mean fluorescence intensity per unit cell area (Supporting Information).

### 4.8 Cell Viability Assay

Displaced cells were collected from the channel outlet, pelleted by centrifugation, resuspended in PBS, and stained with trypan blue. Viability was calculated as the percentage of unstained (live) cells relative to total cells and compared to a control:

$$\%\;\mathrm{viability}=\frac{\#\;\mathrm{live}\;\left(\mathrm{unstained}\right)\;\mathrm{cells}}{\mathrm{total}\;\#\;\mathrm{cells}}\;\times \;100\%$$

## Author Contributions

**Stephen Horan, Anita Wdowicz, Nathan Feely**: methodology, validation, analysis, investigation, visualization, and writing. **Oya Tagit**: investigation and writing. **Wenxin Wang**: methodology. **Gil U Lee**: concept, writing, supervision.

## Conflicts of Interest

The authors declare no conflict of interest.

## Supporting information




**Supporting File**: smll75311‐sup‐0001‐SuppMat1.docx.

## Data Availability

The data that support the findings of this study are available from the corresponding author upon reasonable request.
