# Supplementary material for: Peptide Terminated Polymer Brush Nanomaterials for Label‐Free Separation of Cancer Cells Based on Relative Adhesion
Source: Small. 2026 Aug 19;22(59):e75311. doi: 10.1002/smll.75311 (PMC13690614; doi:10.1002/smll.75311)
Supplement: Supplementary file 1 [file SMLL-22-e75311-s001.docx]

**Supporting Information**

**Peptide terminated brush nanomaterials for label-free separation of cancer cells based on relative adhesion**

Stephen Horan^1^, Anita Wdowicz^1^, Nathan Feely^1^, Peng Li^1^, Oya Tagit^3^, Wenxin Wang^2^ and Gil U Lee^1+^

^1^ School of Chemistry and Conway Institute for Biomolecular and Biomedical Research, University College Dublin, Belfield, Dublin, Republic of Ireland

^2^ Charles Institute, University College Dublin, Belfield, Dublin, Republic of Ireland

^3^ Magnostics Ltd, Monkstown, Co. Dublin, Republic of Ireland

+ Corresponding author: [gil.lee@ucd.ie](mailto:gil.lee@ucd.ie)

1. **Assembly and application of microfluidic device**

**Figure S1** Overview of microfluidic device assembly and application. (A) Image of the assembled microfluidic device. The device consists of six parallel microfluidic channels fabricated in polydimethylsiloxane (PDMS) and chemically bonded to a glass coverslip bearing the polymer brush film. (B) Experimental set-up for cell displacement experiments. Inlet tubing is connected to a syringe pump that drives phosphate-buffered saline (PBS) through the microfluidic channel, displacing cells adhered to the polymer brush film. Cell adhesion and detachment are monitored in real time by brightfield microscopy.

2. **QCM-D analysis of polymer brush growth by ARGET-ATRP**

To characterize ARGET-ATRP polymerization kinetics by quartz crystal microbalance with dissipation (QCM-D), silica QCM-D sensors were functionalized with BiB-APTES as described for glass coverslips (main text, Section 4). The initiator-coated sensors were stabilized in degassed 20% methanol/80% water solution at 50 °C; 60 °C was outside the maximum operating temperature of the QCM-D instrument. Once a stable baseline was established, a pre-heated, degassed polymerization solution prepared under an inert atmosphere — containing PEGMEMA (14.2 g), CuBr_2_ (7.4 mg), 2,2′-bipyridine (Bpy) (15.4 mg), and sodium ascorbate (18 mg) in methanol (8.6 mL) and water (25.5 mL) — was flowed over the sensor at 100 µL min^−1^ and recirculated for the desired polymerization time. The reaction was quenched by replacing the monomer solution with degassed 20% methanol/80% water at 50 °C until a new stable baseline was re-established.

A five-fold molar excess of sodium ascorbate relative to CuBr_2_ was used for QCM-D experiments, contrasting with the 0.5 molar equivalents used for coverslip synthesis (main text). Initial attempts using identical conditions to those established for the coverslips did not yield detectable changes in fundamental frequency or energy dissipation at the end of the experiment, indicating a very low polymerization rate. Stirring efficiency has been previously reported to have a significant effect on the “livingness” and rate of surface-initiated ATRP reactions.^[s1]^ Reduced mixing efficiency within the QCM-D flow cell, relative to the larger stirred reaction vessel used for coverslip synthesis, is postulated to have resulted in elevated local concentrations of the Cu(II) bromide deactivator complex following chain activation, thereby reducing the polymerization rate. A five-fold excess of reducing agent was therefore employed to partially compensate for this effect and achieve a measurable polymerization rate under QCM-D flow-cell conditions.

The frequency and dissipation changes recorded during the QCM-D experiment are presented in Figure S2. Following stabilization of the QCM-D sensor in 20% methanol/80% water solution, the polymerization solution was introduced. An initial sharp drop in Δf was observed due to the change in viscosity of the monomer solution. Upon stabilization in the polymerization solution, a slow, linear decrease in frequency was accompanied by separation of the overtone frequencies and an increase in dissipation. These observations indicate (i) that sensor mass increased linearly with time and (ii) that the polymer brush film exhibits significant viscoelastic damping of the sensor oscillation. The linearity of the frequency decrease is indicative of a well-controlled polymerization, despite the excess of reducing agent employed. It must be noted, however, that the differences in reducing agent concentration and mixing efficiency between the QCM-D and coverslip experiments yielded different growth rates: polymer brush films with a dry thickness of 60 nm were obtained after 270 min of polymerization, corresponding to an approximate growth rate of 0.22 nm min^−1^, significantly lower than the rate of 0.83 nm min^−1^ measured for coverslips using the dedicated polymerization apparatus.

**Figure S2** QCM-D analysis of PEGMEMA brush film growth. (A) Frequency shift (Δf) plot for the 3rd (darkest) to 13th (lightest) overtones, indicating the change in sensor mass. (B) Dissipation (ΔD) plot for the 3rd (darkest) to 13th (lightest) overtones, reflecting the viscoelastic properties of the sensor. For both plots: baseline stabilization, 0–10 min; introduction of polymerization solution and ARGET-ATRP of PEGMEMA brush films, 10–290 min; re-stabilization of baseline, 290–310 min.

***Voigt Model Fitting of QCM-D Data***

The QCM-D experiment was repeated (n = 2) and the compiled data were fitted using a viscoelastic Voigt model in Q-Sense D-Find modelling software (Biolin Scientific, Sweden).^[s2]^ The density of the polymer film was assumed to approximate that of free PEGMEMA monomer at r.t. (1.08 g cm^−3^). The density and viscosity of the bulk solution (20% methanol/water) were set to 0.953 g mL^−1^ and 0.76 mPa·s, respectively. A summary of the modelled polymer brush film properties is presented in Table 1. Voigt model fitting indicates that the PEGMEMA brush film has a shear elastic modulus (G′) of 178.1 kPa and a hydrated thickness of 121.1 nm. Given a dry film thickness of 62.2 nm as measured by ellipsometry, this yields a swelling ratio of 1.9. These values are consistent with those reported by Fu et al., where ARGET-ATRP of oligo(ethylene glycol) methacrylate was studied by QCM-D to yield a shear elastic modulus of ~100 kPa and a swelling ratio of 2.0.^[s3]^

**Table 1** Modelled properties of PEGMEMA brush films determined by Voigt model fitting of in situ QCM-D data.

Despite the agreement between the modelled film properties and previously reported values, interpretations should be made with caution for three reasons. First, the assumed film density (that of free monomer) is an approximation; the actual brush film density will differ from that of the free monomer solution, and this assumption introduces uncertainty into the modelled thickness and modulus. Second, the temperature and solvent conditions under which the properties were measured (50 °C in 20% methanol) do not correlate with those under which the polymer brushes were applied for cell separation (37 °C in PBS). Third, accurate determination of viscoelastic properties of soft nanoscale films by QCM-D is inherently challenging due to the significant damping effects of the film on the crystal resonator, which can introduce artefacts into the extracted moduli.^[s4]^

**References**

[S1] J. B. Kim, W. Huang, M. D. Miller, G. L. Baker, M. L. Bruening, Reversibly cross-linked surface-grafted polymer brushes, J. Polym. Sci. Part A Polym. Chem. 2003, 41, 386.

[S2] M. V. Voinova, M. Rodahl, M. Jonson, B. Kasemo, Viscoelastic acoustic response of layered polymer films at fluid-solid interfaces: Continuum mechanics approach, Phys. Scr. 1999, 59, 391.

[S3] L. Fu, X. Chen, J. He, C. Xiong, H. Ma, Study viscoelasticity of ultrathin poly(oligo(ethylene glycol) methacrylate) brushes by a quartz crystal microbalance with dissipation, Langmuir 2008, 24, 6100.

[S4] Y. Luan, D. Li, T. Wei, M. Wang, Z. Tang, J. L. Brash, H. Chen, "Hearing loss" in QCM measurement of protein adsorption to protein resistant polymer brush layers, Anal. Chem. 2017, 89, 4184.
